# *CmMYB19* Over-Expression Improves Aphid Tolerance in Chrysanthemum by Promoting Lignin Synthesis

**DOI:** 10.3390/ijms18030619

**Published:** 2017-03-12

**Authors:** Yinjie Wang, Liping Sheng, Huanru Zhang, Xinping Du, Cong An, Xiaolong Xia, Fadi Chen, Jiafu Jiang, Sumei Chen

**Affiliations:** College of Horticulture, Nanjing Agricultural University, Nanjing 210095, China; 2013204034@njau.edu.cn (Y.W.); 2013104116@njau.edu.cn (L.S.); zhanghuanru@sina.cn (H.Z.); 2013104106@njau.edu.cn (X.D.); frozen_water@163.com (C.A.); xiaxiaolongyx@163.com (X.X.); chenfd@njau.edu.cn (F.C.); jiangjiafu@njau.edu.cn (J.J.)

**Keywords:** *Chrysanthemum morifolium*, expression analysis, aphid, transgenic plants, lignin

## Abstract

The gene encoding the MYB (v-myb avian myeloblastosis vira l oncogene homolog) transcription factor *CmMYB19* was isolated from chrysanthemum. It encodes a 200 amino acid protein and belongs to the R2R3-MYB subfamily. CmMYB19 was not transcriptionally activated in yeast, while a transient expression experiment conducted in onion epidermal cells suggested that the *CmMYB19* product localized to the nucleus. *CmMYB19* transcription was induced by aphid (*Macrosiphoniella sanborni*) infestation, and the abundance of transcript was higher in the leaf and stem than in the root. The over-expression of *CmMYB19* restricted the multiplication of the aphids. A comparison of transcript abundance of the major genes involved in lignin synthesis showed that *CmPAL1* (phenylalanine ammonia lyase 1), *CmC4H* (cinnamate4 hydroxylase), *Cm4CL1* (4-hydroxy cinnamoyl CoA ligase 1), *CmHCT* (hydroxycinnamoyl CoA-shikimate/quinate hydroxycinnamoyl transferase), *CmC3H1* (coumarate3 hydroxylase1), *CmCCoAOMT1* (caffeoyl CoA *O*-methyltransferase 1) and *CmCCR1* (cinnamyl CoA reductase1) were all upregulated, in agreement with an increase in lignin content in *CmMYB19* over-expressing plants. Collectively, the over-expression of *CmMYB19* restricted the multiplication of the aphids on the host, mediated by an enhanced accumulation of lignin.

## 1. Introduction

The large v-myb avian myeloblastosis vira l oncogene homolog (MYB) transcription factor family is functionally highly diverse. The defining characteristic of these proteins is the presence of the so-called MYB domain, which provides the basis of its DNA binding ability [1]; it comprises some 52 residues which adopt a helix-turn-helix conformation [2]. The MYB proteins have been sub-classified into four types, the largest of which is the R2R3-MYB subfamily [3].

MYB transcription factors are important in the regulation of lignin synthesis, a well-characterized pathway which encompasses the enzymes phenylalanine ammonia lyase (PAL), *c*innamate 4-hydroxylase (C4H), 4-hydroxy cinnamoyl CoA ligase (4CL), hydroxycinnamoyl CoA-shikimate/quinate hydroxycinnamoyltransferase (HCT), p-coumarate 3-hydroxylase (C3H), caffeoyl CoA *O*-methyltransferase (CCoAOMT), cinnamoyl CoA reductase (CCR), ferulate 5-hydroxylase (F5H), caffeic acid/5-hydroxyferulic acid *O*-methyltransferase (COMT) and cinnamyl alcohol dehydrogenase (CAD) [4]. *A. thaliana* MYB46 is not only a key regulator of lignin synthesis, but also activates the entire process of secondary cell wall formation [5]. The over-expression of either *AtMYB58* or *AtMYB63* upregulates a number of lignin synthesis genes, leading to the ectopic deposition of lignin [4]. Similarly, in poplar, the over-expression of *PtoMYB216* activates the transcription of certain lignin synthesis genes, resulting in ectopic lignin deposition [6]. In contrast, the heterologous transcription of *PdMYB221* in *A. thaliana* reduces the cell wall thickness of fibers and vessels, thereby negatively affecting secondary wall formation in the stem [7]. MYB transcription factors typically recognize certain AC-rich *cis* elements ([ACC (T/A) ACC]), which are particularly common in the promoters of *PAL*, *4CL*, *CCR* and *CAD* [8], thereby regulating the lignin biosynthesis.

Aphid is an important insect for crops, which deprives the plant of nutrients and is the vector of certain viruses that further compromise productivity [9]. Plants have evolved a number of strategies to cope with aphid infestation, of which constitutively expressed tolerance is an effective defense in response to insect feeding, which resulted from the presence of host-synthesized compounds or structures, the former including toxins such as tannic acid or mustard oil, and the latter including physical barriers formed by lignin, trichomes and surface wax [10]. We supposed that *MYBs* involved in lignin biosynthesis might regulate constitutive defense to insect feeding by strengthening the physical barrier.

Chrysanthemum (*Chrysanthemum morifolium*) is one of the most valuable ornamental species. One of the major constraints to the quality of the commercial product is the aphid *Macrosiphoniella sanborni*, which greatly hampers chrysanthemum growth and yield [9]. R2R3-MYB genes participate in a wide range of biological processes [11], which includes a few examples of involvement in the response to insect feeding. For example, the *Arabidopsis thaliana* transcription factor *MYB102* restricts the development of *Pieris rapae* caterpillars [12], while *MYB44* activity has been associated with tolerance to both the green peach aphid and the diamondback Moth in Arabidopsis [13]. To date, no *MYB* transcription factors have been associated with the response of chrysanthemum to aphid feeding. Here, a description is given of the isolation of the chrysanthemum *MYB* gene *CmMYB19,* the product of which is associated with lignin synthesis and the defense against aphid feeding.

## 2. Results

### 2.1. Isolation and Phylogenetic Analysis of the CmMYB19 cDNA Sequence

**Figure 1:**
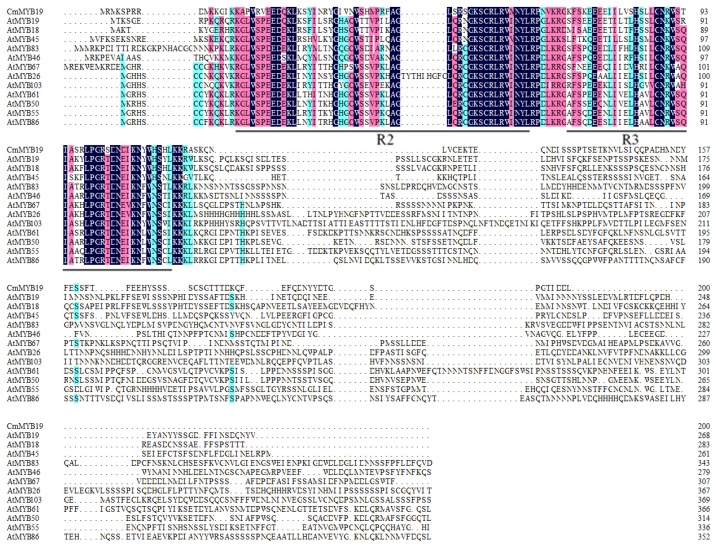
Alignment of the putative CmMYB19 protein sequence with those of R2R3 MYBs from lignin biosynthesis or wall deposition group in Arabidopsis. The two MYB domains are indicated by lines drawn below the alignment.

**Figure 2:**
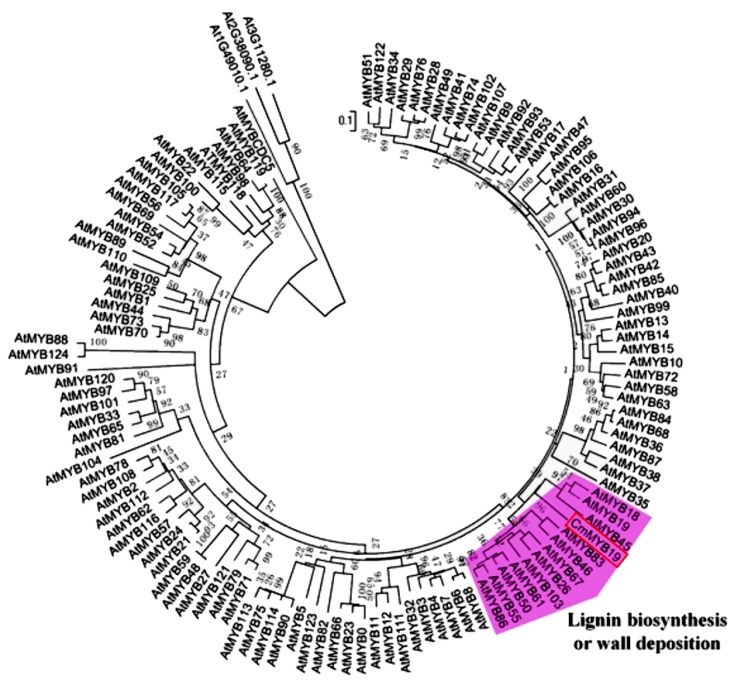
Phylogenetic analysis of CmMYB19 (boxed) and *A. thaliana* R2R3-MYB members. The alignment was generated by ClustalW (available on: http://www.ebi.ac.uk/Tools/msa/ clustalw2/), and the neighbor-joining tree constructed using MEGA 5 (available on: http://www.megasoftware.net) (1000 bootstrap replicates).

The 994 nt *CmMYB19* (KT763375) sequence isolated from NX included a 600 nt ORF. The predicted gene encoded a 200 residue polypeptide containing two conserved MYB domains, on the basis of which it was assigned as a member of the R2R3-MYB subfamily (Figure 1), with a similarity ranging from 31.85% (*AtMYB19*) to 19.30% (*AtMYB103*). An alignment of the deduced amino acid sequences of CmMYB19 with *A. thaliana* R2R3-MYBs showed that *CmMYB19* were included in a lignin biosynthesis group or wall deposition, and its most similar homolog was *AtMYB19* (Figure 2).

### 2.2. Transcriptional Activation and Sub-Cellular Localization

**Figure 3:**
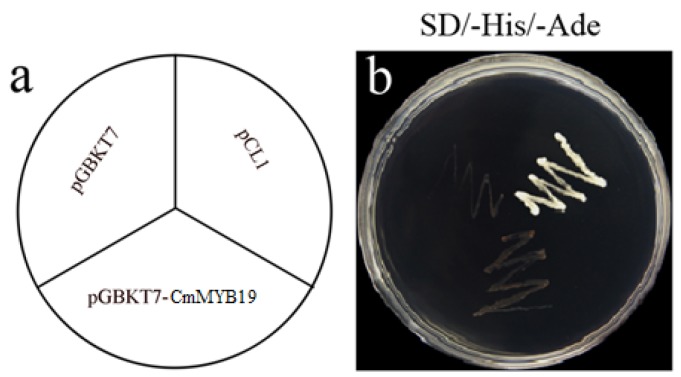
Transcription activation of CmMYB19 in yeast. (**a**) the orientation of the plate. (**b**) the growth of transformed yeast cells. Cells harboring pCL1 were able to grow on the SD/-His-Ade medium (SD, Synthetic Dropout Media), while those containing pGBKT7 could not.

**Figure 4:**
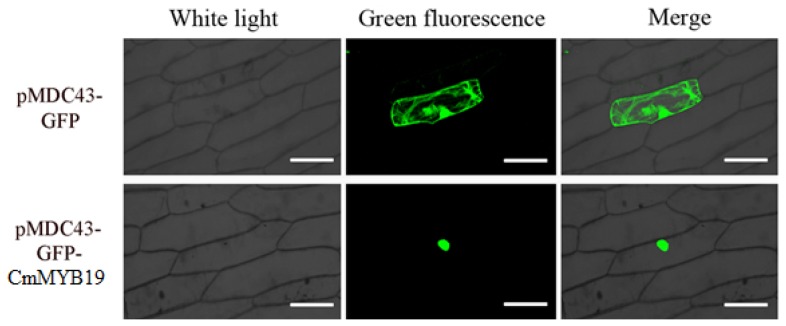
Sub-cellular localization of CmMYB19 expression in transiently transformed onion epidermal cells. Bar: 100 μm.

To characterize the transcriptional activation of CmMYB19. Yeast cells harboring either an empty pGBKT7 or the pGBKT7-CmMYB19 construct were incapable of growing on SD/-His-Ade medium, whereas those harboring pCL1 grew well (Figure 3). Thus, the indication is that CmMYB19 expresses no transcriptional activation in yeast cells. In transiently transformed onion epidermal cells, the control transgene (p35S::GFP) produced a diffuse GFP signal, but the GFP activity induced by the p35S::GFP-CmMYB19 transgene was restricted to the nucleus (Figure 4). Thus, in vivo, CmMYB19 product is likely localized to the nucleus.

### 2.3. Transcription of CmMYB19 in the Chrysanthemum Plant

**Figure 5:**
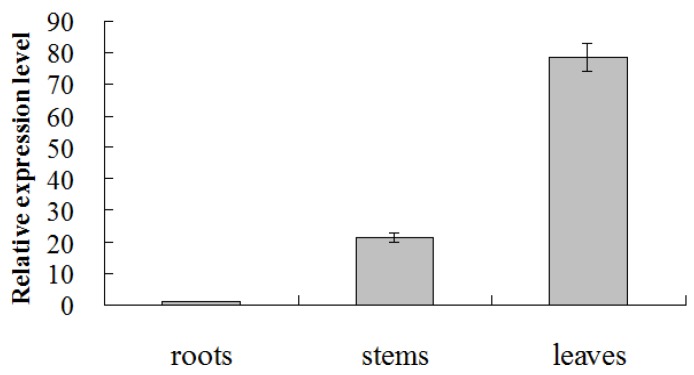
Transcriptional changes of *CmMYB19* in various organs of the chrysanthemum plant, as assayed by qRT-PCR.

**Figure 6:**
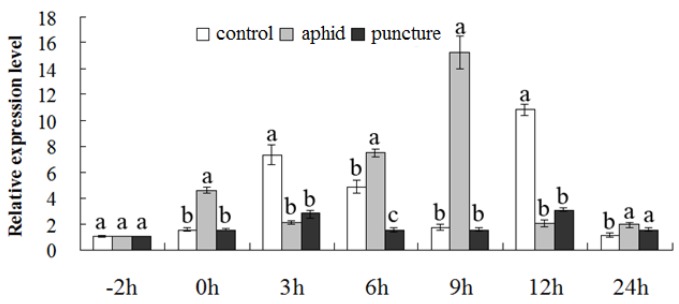
Transcriptional changes of *CmMYB19* following aphid infestation or mock puncture treatment, as assayed by qRT-PCR. At each time point, different letters (a, b, c) designate significantly different expression levels between control, aphid infestation and puncture (*p* < 0.05).

*CmMYB19* transcripts was detectable in the root, stem and leaf of NX plants. The highest abundance was observed in the leaf and the lowest in the root (Figure 5). The pattern of transcription as induced by aphid infestation differed from that induced by the mock puncture treatment. *CmMYB19* was upregulated by the presence of aphids at 0 h, 6 h, 9 h and 24 h, but was downregulated at 3 h and 12 h; meanwhile, the mock puncture treatment lowered the transcript abundance after 3 h, 6 h and 12 h, but had no effect at the other sampling times (Figure 6).

### 2.4. CmMYB19 Over-Expression Enhanced Aphid Tolerance

**Figure 7:**
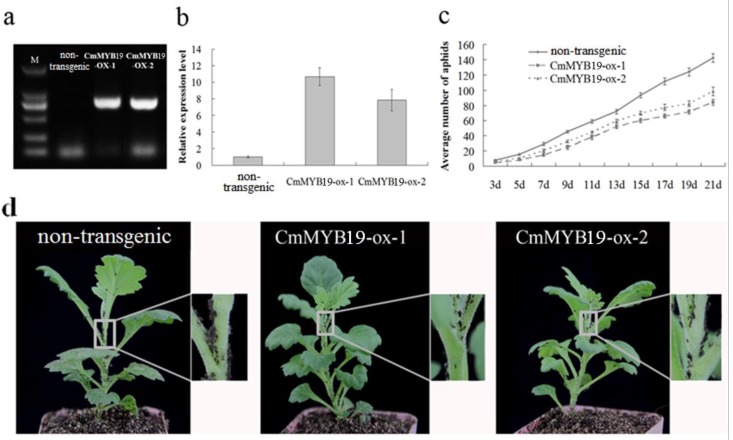
Transgenic chrysanthemum lines over-expressing *CmMYB19* inhibit the multiplication of *M. sanbourni* aphids. (**a**) Amplification of fragment of *Hyg^r^* gene. M, molecular marker, non-transgenic, *CmMYB19*-ox-1, *CmMYB19*-ox-2 were non-transgenic plants and *CmMYB19* overexpressing plants, respectively; (**b**) *CmMYB19* transcript abundance in *CmMYB19* over-expressing and non-transgenic plants; (**c**) the number of aphids present on *CmMYB19* over-expressing and non-transgenic plants measured at 3–21 d (days) after infestation; and (**d**) differential proliferation of aphids between *CmMYB19* over-expressing and non-transgenic plants.

Putative *CmMYB19* overexpressing lines were selected on cultural medium supplemented with hygromycin (Supplemental Figures S1 and S2). The overexpressing lines were verified by amplification of fragment of *Hyg^r^* gene, the bands presented in the transgenic plants while absent in non-transgenic plants (Figure 7a), and *CmMYB19* transcript abundance was clearly higher than in non-transgenic JB in both of the two independent *CmMYB19* over-expression lines (CmMYB19-ox-1 and CmMYB19-ox-2) (Figure 7b). Over the course of the 21 days when aphid numbers were monitored, the aphids multiplied more freely on the non-transgenic plants than on either -ox-1 or -ox-2, whereas, on the non-transgenic plants, the aphids were distributed widely throughout the plants (Figure 7c,d). On the non-transgenic plants, aphid numbers multiplied rapidly from 7 DAI, reaching a mean of 142.4 per plant by 21 DAI (Figure 7c), equivalent to an MR of 28.5 (Table 1). In contrast, on -ox-1, the mean number of aphids present at 21 DAI was only 83.9 (MR = 16.8, IR = 41.1), and the equivalents for -ox-2 were, respectively, 98.4, 19.7 and 30.9 (Figure 7c, Table 1). Thus, the over-expression of *CmMYB19* clearly inhibited the feeding and reproduction of the aphids.

### 2.5. Lignin Content and the Transcript Abundance of Genes Involved in Lignin Synthesis in CmMYB19 Over-Expressors

**Figure 8:**
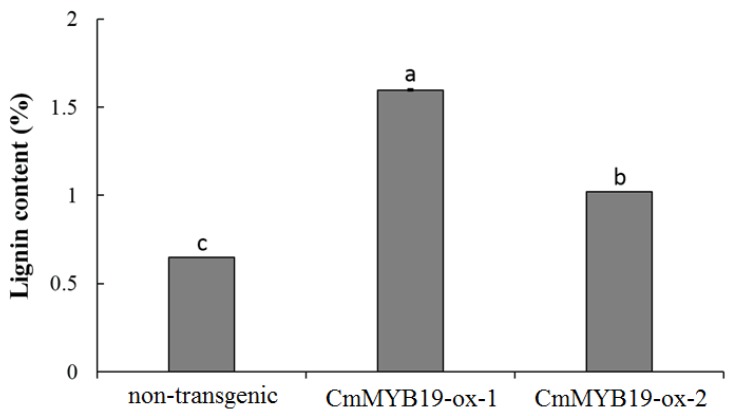
Lignin content determined in *CmMYB19* over-expressing and non-transgenic plants. a, b, c represent significantly different expression levels between non-transgenic and *CmMYB19* overexpressing plants (*p* < 0.05).

**Figure 9:**
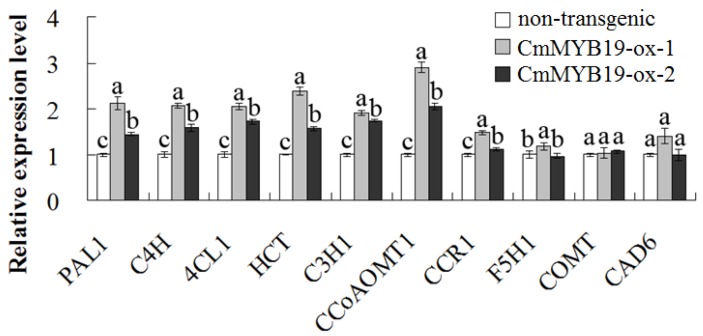
The transcription of genes involved in lignin synthesis in *CmMYB19* over-expressing and non-transgenic plants, as assayed by qRT-PCR. For each gene, different letters (a, b, c) represent significantly different expression levels between non-transgenic and *CmMYB19* overexpressing plants (*p* < 0.05).

The lignin content of both -ox lines was 2.46 and 1.57 fold that of WT JB (Figure 8). Among the lignin synthesis gene surveyed, *CmPAL1*, *CmC4H*, *Cm4CL1*, *CmHCT*, *CmC3H1*, *CmCCoAOMT1* and *CmCCR1* were all more strongly transcribed in the -ox lines, while the abundance of *CmF5H1*, *CmCOMT* and *CmCAD6* transcript was not markedly altered (Figure 9). The conclusion was that *CmMYB19* over-expression promoted the accumulation of lignin by upregulating the transcription of some of the genes involved in lignin synthesis.

## 3. Discussion

Numerous studies have been performed to understand the functions of members of the *MYB* gene family previously [1]. However, rather few data have been generated in chrysanthemum. Two chrysanthemum *MYB* genes have been isolated so far. One of these, *CmMYB1*, when constitutively expressed in *A. thaliana*, has a suppressive effect on tissue lignin content and flavonoid synthesis [14], while the other (*CmMYB2*) not only enhances drought and salinity tolerance, but also increases the plant’s sensitivity to abscisic acid and defers flowering [15]. Here, the R2R3-MYB member *CmMYB19* was shown to be inducible by aphid feeding and appears to contribute to host tolerance against aphid feeding.

The involvement of several *A. thaliana MYB* genes in the host response to aphid feeding have been reported in the literature. These include *AtMYB44*, which regulates tolerance to the green peach aphid and diamondback moth by activating the EIN2-mediated defense pathway [13]. Both *AtMYB15* and *AtMYB38* interact with a harpin protein to modulate the plant’s tolerance to infestation by the green peach aphid [16]. The phylogenetic analysis indicated that CmMYB19 was included in the group of lignin biosynthesis or wall deposition [11], of which *AtMYB103*, *AtMYB61*, *AtMYB55* [17] *AtMYB83*, *AtMYB46* [18], *AtMYB86* [19], *AtMYB26* [20], and *AtMYB50* [21] have been proved to be involved in the biosynthesis of lignin; however, the roles of *AtMYB18*, *AtMYB*19, *AtMYB*45 and *AtMYB*67 in lignin biosynthesis have not been described yet. Here, *CmMYB19* overexpression enhanced the accumulation of lignin. Lignin is a vital component of the vascular plant secondary cell wall and contributes significantly to the erection of a physical barrier against invasion by various pathogens and feeding by various insects [22]. He et al. [23] have shown that the activity of PAL (an enzyme involved in lignin synthesis) is induced in chrysanthemum by aphid feeding, which implies a contribution of lignin to aphid tolerance. Aphids need to access the phloem for nutrient and to the xylem to avoid an excessive build-up of osmotic pressure resulting from their consumption of sugar-rich phloem fluid [24]. Thus, the great lignification of the *CmMYB19* over-expressors may make it more difficult for the aphid stylet to gain access, and thus could act as the brake on aphid multiplication observed in the *CmMYB19* over-expressors.

Lignin synthesis is regulated by a combination of MYB, NAC (*NAM*, *ATAF1/2*, *CUC1/2*) [8] and WRKY named after the highly conserved sequence motif WRKYGQK [25] transcription factors. AtMYB46 and AtMYB83 regulate the lignin pathway by combining with SND1, which holds a closely related NAC domain functioning to activate the entire secondary wall biosynthesis at the top of the network [26,27]. *Populus trichocarpa PtrMYB152* is known to enhance secondary cell wall thickness by elevating lignification activity [28]. The constitutive expression of the *Populus tomentosa MYB* gene *PtoMYB216* in *A. thaliana* activates the expression of a number of lignin synthesis genes, and results in the deposition of lignin even in cells which are normally non-lignified in non-transgenic Arabidopsis [6]. The promoter sequences of both *PAL* and *4CL* feature a high representation of AC elements. The promoters of the two *Eucalyptus gunnii* genes *EgCCR* and *EgCAD2* each harbor a number of conserved MYB binding elements which are crucial for their transcriptional activation in the vascular tissue [29]. In *Eriobotrya japonica*, *EjMYB1* and *EjMYB2* regulate lignification in the fruit via a competitive interaction with AC elements in the promoter region of *Ej4CL1* [30]. The *Populus* (*Populus tremula* × tremuloides) *MYB46* homolog *PttMYB021* activates the transcription in hybrid aspen of lignin synthesis enzymes and mediates the xylem-specific transcription of a number of secondary cell wall carbohydrate-active enzymes through its interaction with AC-type *cis* elements [31]. Here, one yeast hybrid assay suggested that CmMYB19 could bind to AC elements that are rich in lignin biosynthesis genes, suggesting that CmMYB19 functionally act as regulator of lignin biosynthesis (Supplemental Figure S3). Consistently, *CmMYB19* over-expression was shown to upregulate a number of lignin synthesis genes (*PAL1*, *C4H*, *4CL1*, *HCT*, *C3H1*, *CCoAOMT1* and *CCR1*), if AC *cis* elements present in the promoters of any of these genes still remain to be investigated. Most MYB transcription factors appear to possess a transcriptional activation ability, although some only manifest this in the presence of specific bHLH proteins [32]. Here, CmMYB19 appeared to lack transcriptional activity in yeast, so whether CmMYB19 in vivo acts as an activator or whether it regulates lignin synthesis in combination with other protein(s) still need to be established. In the present study, a higher expression level of *CmMYB19* in the leaves and a temporal variation in expression profiles of *CmMYB19* in non-transgenic plants in the absence of aphid infestation such as at time points 3 h and 12 h suggested that *CmMYB19* might participate in other biological processes besides regulating lignin biosynthesis. However, the exact processes are still unknown. Nevertheless, the present study inferred that *CmMYB19* enhanced the aphid tolerance in chrysanthemum via increasing the lignin content. However, the two transgenic lines differed in behaviors of aphid tolerance and the induction of lignin gene expression to some extent, which might result from different spatial integration of *CmMYB19* into the genome. Here, we successfully enhanced the aphid tolerance by modifying the physical defense. Alternative ways, for example, aimed to modify biosynthesis of toxins, and terpenes would obtain higher levels of tolerance or resistance to aphids.

## 4. Materials and Methods

### 4.1. Plant Materials and Growing Conditions

Cuttings of the chrysanthemum varieties “Nannong Xunzhang” (NX: aphid resistant, partially via up-regulating lignin biosynthesis) [33] and “Jinba” (JB: non-resistant) [23] were obtained from the Chrysanthemum Germplasm Resource Preserving Center (Nanjing Agricultural University, Nanjing, China). The cuttings were potted into a 1:2 mixture of vermiculite and garden soil and were maintained in a greenhouse providing a relative humidity of 80% and a 16 h photoperiod given by 100 µmol·m^−2^·s^−1^ of artificial light; the light and dark period temperatures were, respectively, 23 °C and 18 °C.

### 4.2. Isolation and Sequence Analysis of CmMYB19

Total RNA was isolated from NX leaves using the RNAiso reagent (TaKaRa, Tokyo Japan) following the manufacturer’s protocol. A 1 µg aliquot of the resulting RNA was included as the template for first cDNA strand synthesis, using SuperScript III reverse transcriptase (Invitrogen, Carlsbad, CA, USA). The primer pair CmMYB19-M-F/R (Table S1) was designed to amplify a fragment of the *CmMYB19* sequence, based on a previously acquired sequence [33], and RACE (random amplification of cDNA ends) PCR was then used to obtain the full length cDNA. For the 3′-RACE, oligo (dT) was used to synthesize the first cDNA strand, followed by a nested PCR using the adaptor primer (J-R) and CmMYB19-3-1/2 (Table S1). For the 5’-RACE, the AAP and AUAP primers provided with the 5’-RACE System kit v2.0 (Invitrogen) were used in a nested PCR, along with CmMYB19-5-1/2 (Table S1). The PCR products, purified using a Biospin Gel Extraction kit (Bio Flux, Hangzhou, China), were introduced into pMD19-T (TaKaRa) for sequencing. Finally, the *CmMYB19* open reading frame (ORF) was amplified using CmMYB19-F/R (Table S1). The CmMYB19 protein sequence was aligned with its homologs using ClustalW software (available on: http://www.ebi.ac.uk/Tools/msa/ clustalw2/) [34] and a phylogenetic tree was generated using MEGA 5 software (available on: http://www.megasoftware.net) [35] based on the neighbor-joining method and 1,000 bootstrap replicates following previous descriptions [11,36]. *A. thaliana* MYB polypeptides sequences were obtained from the Plant TFDB website (available on: planttfdb.cbi.edu.cn/).

### 4.3. Transcriptional Activation Assay and Sub-Cellular Localization of CmMYB19

The *CmMYB19* ORF was amplified using a Phusion^®^ High Fidelity PCR Kit (New England Biolabs, Ipswich, MA, USA), and *Kpn*I and *Xho*I restriction sites were introduced with the primer pair CmMYB19-KPN-F/-XHO-R (Table S1). The resulting amplicon was digested by *Kpn*I/*Xho*I, then ligated into pENTR™1A (Invitrogen) to form the construct *pENTR™1A-CmMYB19*, which acts as a donor construct for yeast expressing construct and binary vector for chrysanthemum transformation. A number of clones were sequenced for validation purposes. For transcription activation ability assay of CmMYB19, GAL4/UAS system was used, where *CmMYB19* was fused to GAL4 DNA biding domain using pDEST-GBKT7 vector [37]. To generate yeast expressing a construct pDEST-GBKT7-*CmMYB19*, *CmMYB19* sequence was inserted into pDEST-GBKT7 via an LR (LR Clonase™ II enzyme mix (Invitrogen) reaction between pENTR™1A-*CmMYB19* and pDEST-GBKT7. The resulting construct pDEST-GBKT7-*CmMYB19*, pDEST-GBKT7 (negative control) and *pCL1* (positive control) were each introduced separately into Y2HGold yeast cells (Clontech, Mountain View, CA, USA), following the manufacturer’s protocol [38]. Transformants harboring either pDEST-GBKT7-*CmMYB19* or pDEST-GBKT7 were selected on SD/-Trp medium (SD, Synthetic Dropout Media), while those harboring *pCL1* were selected on SD/-Leu medium. Selected clones were then transferred to an SD/-His-Ade medium and incubated for two to three days at 30 °C, transcription activity was determined when the clones could induce expression of reporter genes and grow on the SD/-His-Ade medium. The sub-cellular localization of CmMYB19 was identified by transiently transforming onion epidermal cells with the construct p35S::GFP-*CmMYB19* (GFP, the green-fluorescent protein) generated by LR reaction between pENTR™1A-*CmMYB19* and pMDC43 (Invitrogen) [39]. The p35S::GFP-*CmMYB19* construct and the empty pMDC43 vector were introduced into onion epidermal cells via particle bombardment (PDS-1000; Bio-Rad, Tokyo, Japan). Transformed cells were held for 16 h on Murashige and Skoog (MS) medium in the dark, and then subjected to confocal laser microscopy in order to monitor GFP activity [40].

### 4.4. Transcription of CmMYB19 and Its Response to Aphid Infestation

Roots, stems and leaves were harvested from four week old NX plants to characterize the topological profile of *CmMYB19* transcription. To investigate the response to aphid (*M. sanborni*) feeding, a phenotyptically uniform set of 8–10 leaf stage NX plants were each infested with 20 s instar nymphs. The nymphs were placed onto the third fully expanded leaf (counting from the stem apex) using a soft brush. The inoculated leaves were enclosed within a transparent, ventilated plastic cage. The leaves of three inoculated plants were harvested at −2 h, 0 h, 3 h, 6 h, 9 h, 12 h, 24 h (where 0 h refers to 2 h after the infestation), following Xia et al. [33]. As a control to account for any stress imposed by aphid stylet penetration, a mock puncture treatment was imposed, in which leaves were pricked with a needle five times at 0 h, ten times at both 3 h and 6 h, 15 times at 9 h and 12 h and 20 times at 24 h [41]. Each experiment was replicated three times. Leaf samples required for RNA extraction were snap-frozen in liquid nitrogen and stored at −80 °C. Total RNA (isolated as described earlier) was treated with RNase-free DNase I (TaKaRa), and then 1 µg treated RNA was converted into cDNA using M-MLV (MLV, clone 3 Moloney-murine leukemia virus) reverse transcriptase (TaKaRa) following the manufacturer’s protocol. Transcript abundance was detected by quantitative real-time PCR (qRT-PCR). The reactions contained SYBR^®^ Premix Ex Taq™ II (Tli RNaseH Plus) (TaKaRa) and the primer pair CmMYB19-RT-F/R (Table S1). The primer pair CmEF1á-F/R was used to amplify the reference gene *CmEF1á*. Fold changes were calculated using the 2^−ΔΔ*C*t^ method [42]. The experiment included three biological replicates. The statistics was calculated from three biological replicates.

### 4.5. Generation of Transgenic Chrysanthemum and the Determination of Aphid Tolerance

*CmMYB19* over-expressors in a JB background were generated by leaf disc agroinfection, based on *Agrobacterium tumefaciens* strain EHA105 [43]. RNA was extracted (as described in an earlier section) from both putative *p35::CmMYB19*-harboring and WT (the non-transgenic chrysanthemum ‘Jinba’) plants. *CmMYB19* transcript abundance was detected using qRT-PCR. Five second instar *M. sanborni* nymphs were placed on both WT plants and two independent over-expressing lines. Each plant was then enclosed in a 25 cm × 12 cm transparent plastic cylinder capped with gauze [38]. Aphid numbers were counted every two days starting three days after the initial aphid infestation (DAI) and ending after 21 days. The experiment involved three replicates of 20 plants per each tested line. The aphid numbers were used to calculate both a multiplication rate (MR) and an inhibition ratio (IR), where MR was given by the number of aphids present at 21 DAI divided by five. IR was calculated from the ratio (*N*_W_ − *N*_T_)/*N*_W_, where *N*_W_ and *N*_T_ represented the number of aphids present at 21 DAI on, respectively, WT and the over-expressing plants.

### 4.6. Determination of Lignin Content and the Transcription of Lignin Synthesis Genes

For lignin content analysis, stems from WT and *CmMYB19* over-expressing plants at the 6–8 leaf stage were harvested, lignin content was determined using the acetyl bromide method [44]. Briefly, harvested stems were dried at 60 °C for 48 h. In addition, 20 mg dried samples were sonicated with 5 mL acetone for 30 min in a 10-mL screw-capped test tube. The supernant extract was pipetted off, and the extractive free sample was dried, and redissolved in 5 mL 20% (*v*/*v*) AcBr-acetic acid solution containing 100 µL 70% perchloric acid and the sample was kept in a block heater at 50 °C for 3 h with regular shaking. The reaction was stopped by keeping the samples at −20 °C for 15 min. The solution was transferred to a 5-mL screw-capped test tube containing 1 mL 2 M NaOH and 0.1 mL freshly prepared 0.5 M hydroxylamine hydrochloride, filled up to 5 mL with glacial acetic acid, and inverted several times in order to mix. The UV spectrum was measured with a Shimadzu UV-2401 spectrometer (Shimadzu Corp, Kyoto, Japan) at 280 nm. Lignin content was calculated using the following expression: Lignin% = 100 (As − Ab) V/*a*W, where As represents absorbance of sample; Ab is absorbance of blank; V represents the volume of solution; W is weight of sample; and *a* is the absorptivity of a lignin standard calculated for each analysis series. The transcript abundances of *CmPAL1*, *CmC4H*, *Cm4CL1*, *CmHCT*, *CmC3H1*, *CmCCoAOMT1*, *CmCCR1*, *CmF5H1*, *CmCOMT* and *CmCAD6* were detected using qRT-PCR, as described above, with relevant primer sequences given in Table S1. The template for the qRT-PCRs was total RNA prepared, as described above, from the stems of both WT and *CmMYB19* over-expressing plants. The relative expression level of a gene is the fold change in expression of a gene in a sample compared to the calibrator sample. The expression level of *CmMYB19* in the root, that at “−2 h” time point of control, and of non-transgenic plants, were set as calibrator “1”, respectively. The experiment included three biological replicates.

### 4.7. Statistical Analysis

Tukey’s multiple range test (*p* = 0.05) was used to compare means. Calculations were performed by routines implemented in SPSS 16.0 software (SPSS Inc., Chicago, IL, USA).

## 5. Conclusions

Taken together, MYB transcription factor CmMYB19 from chrysanthemum encodes a R2R3-MYB. It localized in the nuclei, and expresses no transcriptional activation in yeast cells. CmMYB19 transcription was induced by aphid infestation. The over-expression of CmMYB19 restricted the multiplication of the aphids on the host, mediated by an enhanced accumulation of lignin.

## Figures and Tables

**Table 1:** Aphid multiplication on *CmMYB19* over-expressing and non-transgenic plants

Plants		MR *	IR
Non-transgenic plants		28.48 ± 0.262 ^a^	0
Transgenic plants	CmMYB19-ox-1	16.78 ± 0.548 ^c^	41.08
	CmMYB19-ox-2	19.68 ± 0.776 ^b^	30.90

* different letters (^a^, ^b^, ^c^) represent significantly different aphid multiplication between non-transgenic and CmMYB19 overexpressing plants.

## Supplementary Materials

Supplementary materials can be found at www.mdpi.com/1422-0067/18/3/619/s1.
