# Supplementary material for: CmMYB19 Over-Expression Improves Aphid Tolerance in Chrysanthemum by Promoting Lignin Synthesis
Source: Int J Mol Sci. 2017 Mar 12;18(3):619. doi: 10.3390/ijms18030619 (PMC5372634; doi:10.3390/ijms18030619)
Supplement: Supplementary file 1 [file ijms-18-00619-s001.pdf]

Table S1. Primer sequences used in the present study.

Primer	Sequence (5′-3′)	remark
Oligo(dT)	GACTCGAGTCGACATCGATTTTTTTTTTTTTTTTT	RT-PCR
CmMYB19-M-F	GCATCTGGAACTGGTCTCACA	
CmMYB19-M-R	TCAGATGTTGGTGACGAGGA	
CmMYB19-3-1	CCTTCTCGGAAATAGGTGGTCT	3′RACE
CmMYB19-3-2	TCCCATCTGAAGAAACGAGCA	
J-R	CTGATCTAGAGGTACCGGATCC	
CmMYB19-5-1	TTCAGATGGGAATGCCAATAGTT	5′RACE
CmMYB19-5-2	CGAGAAGGGAATGAGAAACAAGTATG	
AAP	GGCCACGCGTCGACTAGTACGGGIIGGGIIGGGIIG	
AUAP	GGCCACGCGTCGACTAGTAC	
CmMYB19-F	AGTAACGTACTAGCAATTGGAACTAAGT	Full-length
CmMYB19-R	ATTAAAGATCGTCAATGGTTCCTG	PCR
CmMYB19-KPN-F	CGGGGTACCGAATGAGAATGAAGTCTCCAAGAAGGG	Digestion sites
CmMYB19-XHO-R	CCGCTCGAGTGAAGATCGTCAATGGTTCCTGGTG	insertion
CmMYB19-RT-F	ACATTTCCTCGTCACCAACATCT	qRT-PCR
CmMYB19-RT-R	GAAACTCAAACTGCTTGTCTGTGGT	
CmPAL1-F	CCCCAACAGGATCAAGGCAT	
CmPAL1-R	TTGTCGAACTCTTCACCCGG	
CmC4H-F	CTCCAAACTTCGCGGCAAAA	
CmC4H-R	AGTTACGTTGACCCATGCGT	
Cm4CL1-F	TTCATCGTTGACCGGCTCAA	
Cm4CL1-R	TCCTCCGTCAAACTTGAGCC	
CmHCT-F	GCCTATAGCAGTAGCCGGTG	
CmHCT-R	CTTTGCCAAAGCGTCGTGAA	
CmC3H1-F	CGCCAATGTCAAGGTTGGTG	
CmC3H1-R	GTGGCAGGATCTCGAGCTAC	
CmCCoAOMT1-F	TGGCTGCCGATCCAAGAATT	
CmCCoAOMT1-R	GACTCGACGGCAAAGGGTAA	
CmCCR1-F	CATTTGTGTCACCGGTGCTG	
CmCCR1-R	AACGGTTCCTCGAACAGCAT	
CmF5H1-F	CTTCATTGACCCCGCTGGAT	
CmF5H1-R	TTTCGGCATCTTCATCGCCT	
CmCOMT-F	TGTCATGACTGGAGTGACGC	
CmCOMT-R	GGAAGAATGCATTCCGCGAC	
CmCAD6-F	CCGATGGAATGTCTCCCGAG	
CmCAD6-R	ACGTGATGTCCCATCGCTTT	
Hyg-F	CGACAGCGTCTCCGACCTGAT	
Hyg-R	AGATGTTGGCGACCTCGTATTG	

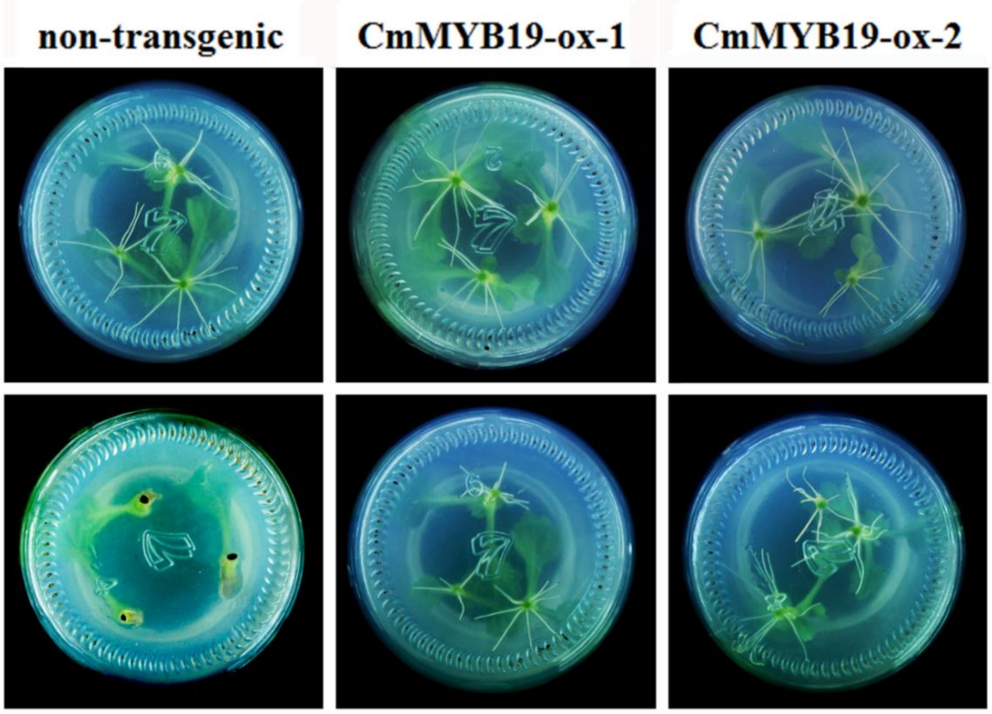

Figure S1. Rooting selection assay with and without hygromycin for putative transgenic and non-transgenic plants. Upper panel, MS medium without hygromycin; Lower panel, MS medium supplemented with hygromycin.

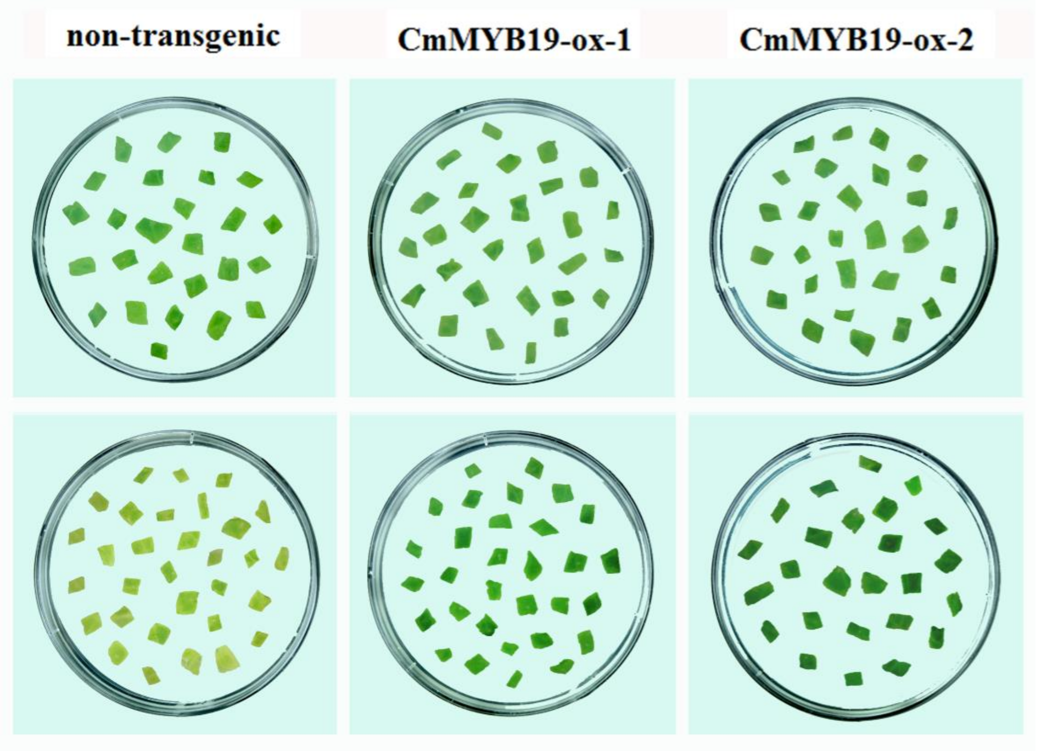

Figure S2. Regeneration assay with and without hygromycin selection for putative transgenic and non-transgenic plants. Upper panel, MS medium without hygromycin; Lower panel, MS medium supplemented with hygromycin. The photos were taken at 10d after inoculation.

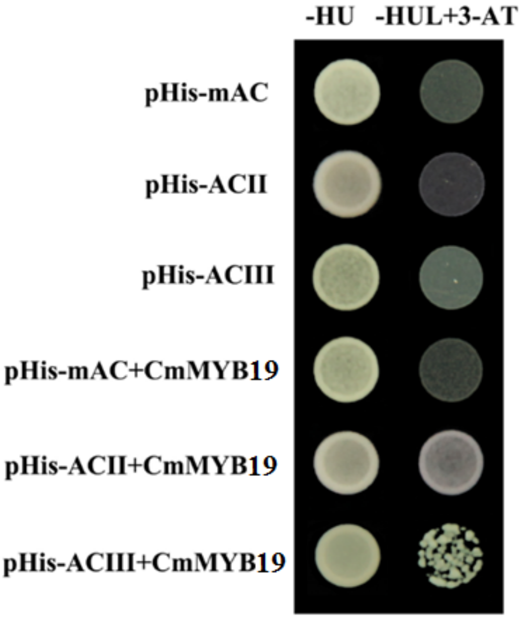

Figure S3. Yeast one hybrid assay for binding ability of *CmMYB19* to AC element.
